# Sertoli-Leydig Cell Tumour With Heterologous Elements Presenting as Hyperandrogenaemia and Markedly Elevated Alpha-Fetoprotein: A Challenging Diagnosis

**DOI:** 10.7759/cureus.116833

**Published:** 2026-09-24

**Authors:** Baraa Albousen, Joseph Mechery

**Affiliations:** 1 Obstetrics and Gynaecology, Ysbyty Glan Clwyd Hospital, Rhyl, GBR

**Keywords:** alpha-fetoprotein, diagnostic delay, dicer1, heterologous elements, hyperandrogenaemia, polycystic ovarian syndrome, sertoli-leydig cell tumour, sex cord-stromal tumour

## Abstract

Sertoli-Leydig cell tumours (SLCTs) are rare ovarian sex cord-stromal tumours that classically present with features of virilisation due to androgen excess and are frequently misdiagnosed as polycystic ovarian syndrome (PCOS).

We report a case of a 33-year-old woman with a three-year history of progressive hirsutism, weight gain, and secondary amenorrhoea, initially attributed to PCOS. An incidental pelvic ultrasound arranged in the context of an endocrinology review revealed a large complex solid-cystic mass measuring 21.2 × 13.8 × 23.4 cm. Tumour markers demonstrated a markedly elevated alpha-fetoprotein (AFP) of 8,147 KU/L, raising initial suspicion of a yolk sac (germ cell) tumour. CT imaging confirmed a 23 cm predominantly cystic pelvic mass with no evidence of metastatic disease. Following multi-disciplinary team discussion and referral to a specialist germ cell tumour MDT, the patient underwent laparotomy and left salpingo-oophorectomy. Initial histology reported an immature teratoma; however, following expert second-opinion review by Professor McCluggage, the final diagnosis was revised to a moderately to poorly differentiated SLCT with heterologous intestinal-type mucinous elements and a small carcinoid component. Ascitic fluid cytology demonstrated no malignant cells.

This case highlights the risk of anchoring bias in attributing hyperandrogenaemia to PCOS, the capacity of SLCT with heterologous mucinous elements to mimic germ cell tumours biochemically and histologically, and the indispensable role of expert pathological review in rare ovarian tumours.

## Introduction

Sertoli-Leydig cell tumours (SLCTs) are uncommon ovarian sex cord-stromal neoplasms that can pose diagnostic and management difficulties, typically presenting with androgenic features in women in their second to third decade of life [1]. Clinically, virilisation develops in a stepwise fashion, beginning with defeminising features such as amenorrhoea and breast atrophy before progressing to masculinising signs including hirsutism, clitoromegaly, and voice deepening. Because these features overlap closely with polycystic ovarian syndrome (PCOS), SLCT is frequently misdiagnosed as PCOS, particularly when presentation is gradual.

Marked elevation of alpha-fetoprotein (AFP) is an unusual finding in SLCT, yet among non-germ cell ovarian tumours, it remains the one most frequently associated with AFP production [2]. When heterologous elements are present, this can create substantial diagnostic overlap with germ-cell tumours both biochemically and histologically, as both entities can raise AFP and share overlapping immunohistochemical markers. SLCTs account for less than 0.5% of all primary ovarian tumours, making them a rare entity even among sex cord-stromal neoplasms, which themselves comprise only around 8% of ovarian tumours overall [1,2].

"Heterologous elements" refers to tissue types foreign to the ovary that can arise within these tumours, most often intestinal-type mucinous glands, but occasionally hepatocytic, cartilaginous, or rhabdomyosarcomatous tissue. When present, they are associated with a less favourable prognosis and, as in this case, additional diagnostic complexity. Standard management is surgical, with the extent of surgery (fertility-sparing unilateral salpingo-oophorectomy versus more radical resection) individualised according to age, fertility wishes, and stage; adjuvant chemotherapy is generally reserved for higher-risk disease given limited evidence of benefit in early-stage tumours.

We report an illustrative case highlighting this diagnostic overlap and the value of expert pathological review in reaching the correct diagnosis.

## Case presentation

Initial presentation and investigations

We report the case of a 33-year-old woman who presented with a three-year history of worsening hirsutism, significant weight gain, and secondary amenorrhoea. Primary care investigations in 2025 identified a raised testosterone (6.3 nmol/L) and raised thyroid-stimulating hormone (TSH) (9.3 mU/L); hence, she was referred to an endocrinologist. She had a pelvic scan, which revealed a large complex solid lesion measuring 21.2 × 13.8 × 23.4 cm, extending above the umbilicus with no vascularity. Neither ovary was separately identifiable, and there was no free fluid (Figure 1A). Colour Doppler confirmed absent intrinsic vascularity (Figure 1B). She was referred to gynaecology. On review, she had a large pelvic-abdominal mass of 36 to 40 weeks' size. Tumour marker tests showed AFP 8147 KU/L, CA 125 78 KU/L, CA 19.9 7 KU/L, CEA 2 microg/mL, and hCG <1 IU/L (Table 1). CT scan of the thorax, abdomen, and pelvis revealed a large complex, predominantly cystic pelvic mass measuring 23 cm, with solid enhancing components extending into the upper abdomen. There was no peritoneal or metastatic disease. Incidental left renal calculi were noted. The clinical timeline of presentation, investigation, and management is summarised in Figure 2. She was discussed at the gynaecology oncology MDT and then at the germ cell tumour MDT at the specialist centre.

**Figure 1:**
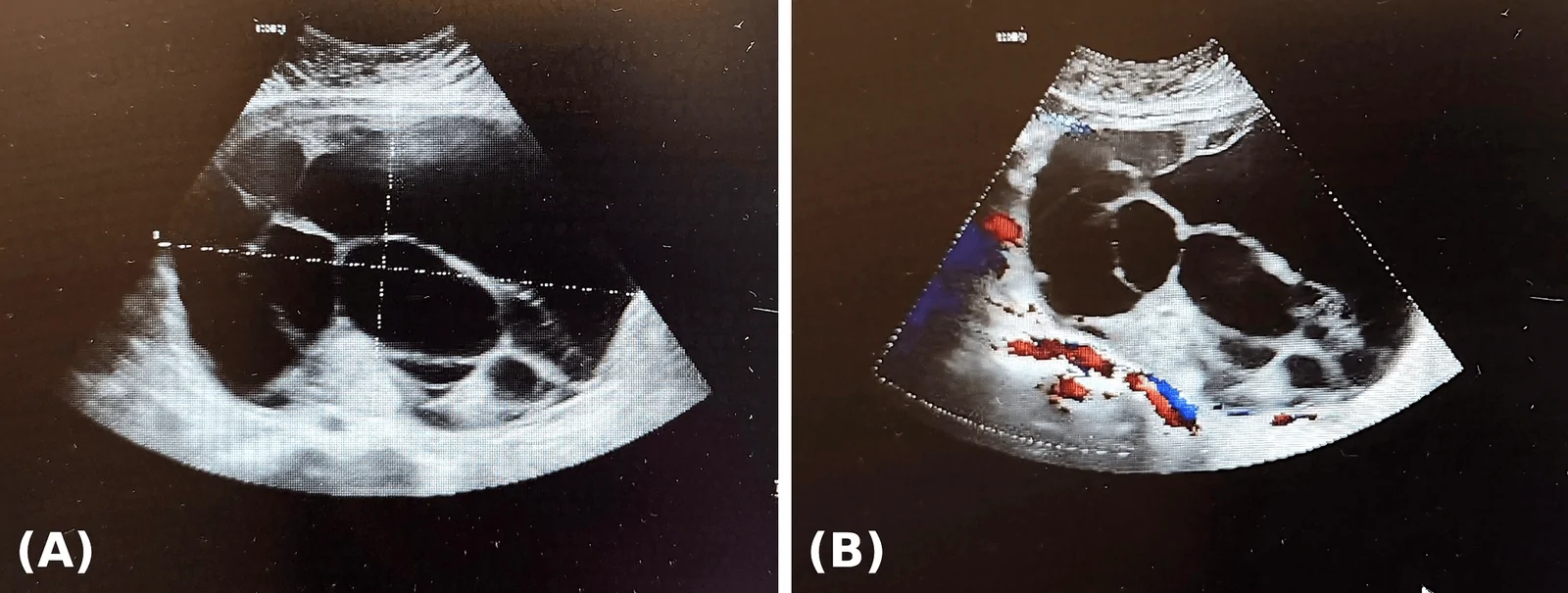
Pelvic ultrasound (Day 1) showing a large complex left ovarian mass (measuring 21.2 × 13.8 × 23.4 cm) with mixed solid (echogenic) and cystic (anechoic) components (A) B-mode demonstrating the solid-cystic architecture, with measurement callipers across the mass. (B) Colour Doppler showing absent intrinsic vascularity within the mass.

**Table 1:** Tumour markers at presentation

Investigation	Result	Reference Range
Alpha-fetoprotein (AFP)	8,147 KU/L (High)	H 1-6 KU/L
CA-125	78 KU/L (High)	<35 KU/L
CA19-9	7 KU/L	<35 KU/L
CEA	2 µg/L	<4 µg/L
Beta-HCG	<1 IU/L	<1 IU/L
Serum testosterone	6.3 nmol/L (High)	0.5-2.6 nmol/L
Thyroid-stimulating hormone	9.3 mU/L (High)	0.4-4.0 mU/L

**Figure 2:**
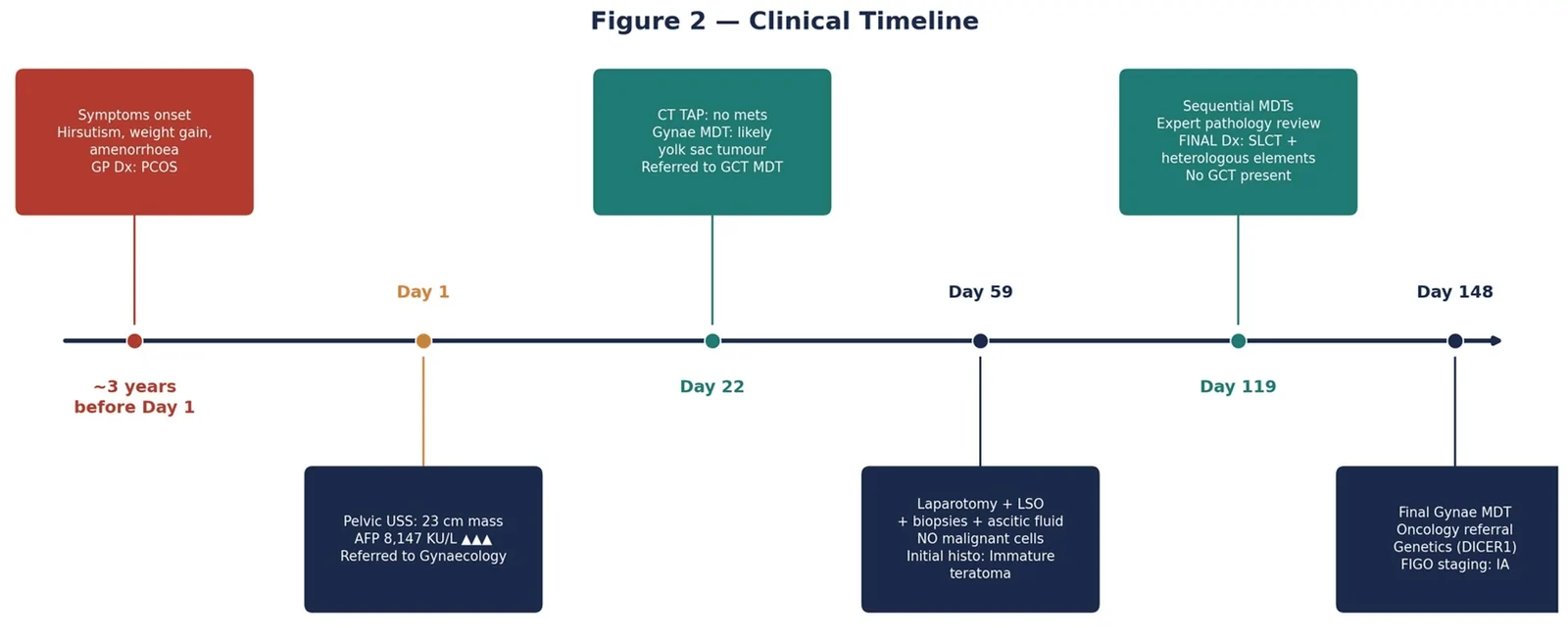
Clinical timeline from symptom onset to final multidisciplinary management plan

Surgical management

As per specialist centre recommendation, she underwent laparotomy, left salpingo-oophorectomy, infracolic omentectomy, peritoneal biopsy, and peritoneal fluid collection for cytology. The excised ovarian mass measured 280 mm on gross pathological examination, with a multiloculated cystic and solid cut surface; the capsule was intact at the time of surgical removal, with capsular rupture occurring subsequently during specimen handling, and this was staged as FIGO IA [3]. As this was a fertility-conserving unilateral procedure, the contralateral ovary and uterus were preserved.

Histopathology and follow-up plan

Initial histology reported an immature teratoma with neuroectodermal elements; however, given diagnostic uncertainty regarding the differential - which included mucinous adenocarcinoma, carcinosarcoma, and sex cord-stromal tumour with heterologous elements of intestinal type - the case was referred for expert second-opinion review. This confirmed an SLCT of moderate to poor differentiation, with heterologous elements: abundant heterologous intestinal-type mucinous glands, in keeping with a mucinous borderline-type appearance but arising within the sex cord-stromal tumour rather than representing a true mucinous borderline neoplasm, together with a small heterologous carcinoid component. Tumour cells were mitotically active; formal mitotic count, necrosis, and lymphovascular invasion status were not specified in the original pathology report. Immunohistochemistry showed the intestinal-type glandular elements diffusely positive for CDX2 and focally positive for SALL4 and glypican-3, accounting for the AFP elevation; the sex cord-stromal component was positive for inhibin, calretinin, and WT1; and the carcinoid component was diffusely positive for synaptophysin, CD56, and chromogranin. No germ cell tumour was present, and ascitic fluid cytology showed no malignant cells. She was again discussed at the gynaecology oncology MDT and referred for adjuvant chemotherapy. A genetic referral was also made to test DICER1; results are pending at the time of reporting.

Post-operative oncological follow-up

At oncology review 14 weeks post-surgery, tumour markers had normalised (AFP 1 KU/L, CA-125 17 KU/L, testosterone 1.3 nmol/L, TSH 2.40 mU/L), and interval CT imaging showed no evidence of recurrence. Adjuvant BEP chemotherapy was discussed, including its toxicities, but declined following shared decision-making, given the limited evidence base in Stage IA SLCT and an estimated 80%-90% five-year survival with surgery alone [4]; active surveillance with three-monthly review was adopted instead. A new contralateral ovarian cyst seen on interval CT was considered likely benign, and oncological follow-up was subsequently stood down, with surveillance transferred to the gynaecological team. At the time of reporting, follow-up extends to approximately five months post-surgery, with tumour markers remaining normal throughout and a further gynaecological review scheduled to reassess the contralateral cyst.

## Discussion

SLCTs are uncommon ovarian neoplasms that mainly arise in reproductive-age women, with a reported mean age at presentation of around 25 years [1]. Most cases occur sporadically, although a subset arises in the context of DICER1 syndrome; somatic or germline DICER1 mutations have been reported in up to roughly 65% of SLCTs [2].

Androgenic features are seen in a majority of cases and are more common with moderate or poor tumour differentiation [5]; as differentiation worsens, signs of virilisation become more likely, often beginning with defeminisation [6] and progressing to masculinisation [7]. Our patient displayed both defeminising and masculinising features, consistent with direct androgen secretion by the tumour, which accounts for her raised testosterone and hirsutism.

In this case, the markedly elevated AFP most likely reflected the heterologous intestinal-type mucinous glands within the tumour, which can produce AFP and stain positively for SALL4 and glypican-3 - markers otherwise used to support a germ cell tumour diagnosis, explaining the initial diagnostic overlap [8].

SLCTs are graded by the WHO as well-, moderately, or poorly differentiated. Standardised treatment guidance is lacking, but surgical resection remains the mainstay, with postoperative chemotherapy considered on a case-by-case basis.

Overall prognosis is favourable, though outcome correlates closely with stage and differentiation [6]; adjuvant chemotherapy is generally reserved for advanced stage, intermediate-to-poor differentiation, retiform pattern, or heterologous elements [9]. For Stage IA disease specifically, published outcomes suggest a five-year survival of approximately 80%-90% with surgery alone [4], which supported our patient's shared decision to pursue active surveillance rather than adjuvant chemotherapy despite the presence of heterologous elements.

Poorer differentiation, advanced stage, and mesenchymal heterologous elements are each associated with higher recurrence and reduced survival, with five-year survival falling to roughly 77.8% in higher-grade tumours and 33.3% in advanced-stage disease [10]. While most relapses occur within the first two years, later recurrence - including in the contralateral ovary - has been reported [11]; given this and the limited evidence for adjuvant chemotherapy in Stage IA disease, our patient chose active surveillance over adjuvant treatment after detailed shared decision-making.

## Conclusions

Diagnosing SLCT is challenging, particularly in tumours with heterologous elements, which can mimic germ-cell tumours both biochemically, through AFP elevation, and histologically. This case underscores the value of expert pathological review in reaching an accurate diagnosis and guiding appropriate management. Surgical resection remains the mainstay of treatment; adjuvant chemotherapy may be considered in higher-risk disease, though its benefit in Stage IA tumours is not well established, and its use should be individualised. Prognosis in early-stage SLCT is generally favourable, but this varies with stage, differentiation, and the presence of heterologous elements, and our patient's short follow-up to date is insufficient to confirm long-term disease control. In the absence of standardised treatment guidelines, management should follow a multidisciplinary approach with ongoing surveillance.
